# The association of maternal psychosocial stress with newborn telomere length

**DOI:** 10.1371/journal.pone.0242064

**Published:** 2020-12-10

**Authors:** Monika A. Izano, Lara J. Cushing, Jue Lin, Stephanie M. Eick, Dana E. Goin, Elissa Epel, Tracey J. Woodruff, Rachel Morello-Frosch

**Affiliations:** 1 Program on Reproductive Health and the Environment, Department of Obstetrics, Gynecology and Reproductive Sciences, University of California, San Francisco, CA, United States of America; 2 Department of Environmental Health Sciences, Fielding School of Public Health, University of California, Los Angeles, CA, United States of America; 3 Department of Biochemistry and Biophysics, University of California, San Francisco, CA, United States of America; 4 Department of Psychiatry, University of California, San Francisco, CA, United States of America; 5 Department of Environmental Science, Policy and Management and School of Public Health, University of California, Berkeley, CA, United States of America; University of Newcastle, UNITED KINGDOM

## Abstract

**Background:**

Telomere length in early life predicts later length, and shortened telomere length among adults and children has been linked to increased risk of chronic disease and mortality. Maternal stress during pregnancy may impact telomere length of the newborn.

**Methods:**

In a diverse cohort of 355 pregnant women receiving prenatal and delivery care services at two hospitals in San Francisco, California, we investigated the relationship between self-reported maternal psychosocial stressors during the 2^nd^ trimester of pregnancy and telomere length (T/S ratio) in newborn umbilical cord blood leukocytes. We examined financial strain, food insecurity, high job strain, poor neighborhood quality, low standing in one’s community, experience of stressful/traumatic life events, caregiving for a dependent family member, perceived stress, and unplanned pregnancy. We used linear regression and Targeted Minimum Loss-Based Estimation (TMLE) to evaluate the change in the T/S ratio associated with exposure to each stressor controlling for maternal age, education, parity, race/ethnicity, and delivery hospital.

**Results:**

In TMLE analyses, low community standing (-0.09; 95% confidence interval [CI]-0.19 to 0.00) and perceived stress (-0.07; 95% CI -0.15 to 0.021 was marginally associated with shorter newborn telomere length, but the associations were not significant after adjusting for multiple comparisons. All linear regression estimates were not statistically significant. Our results also suggest that the association between some maternal stressors and newborn telomere length varies by race/ethnicity and infant sex.

**Conclusions:**

This study is the first to examine the joint effect of multiple stressors during pregnancy on newborn TL using a flexible modeling approach.

## Introduction

Telomeres are DNA-protein complexes that cap the end of chromosomes to protect the cell’s genomic stability; shortened telomere length in white blood cells has been linked to increased risk of chronic disease and mortality [1]. Prior research has inferred that telomere length in proliferative and minimally proliferative tissues is established in early life and that stem cells of these tissues replicate at similar rates during adulthood [2]. Thus, it is hypothesized that telomere length at birth may be an important predictor of telomere length later in life [2].

Genetic and environmental factors start exerting their effects on telomere attrition as early as in utero and extend throughout the lifespan [3–5], thereby potentially creating a long-term trajectory that contributes to individual susceptibility for chronic diseases. Maternal stress during pregnancy has been associated with shorter newborn telomere length measured in umbilical cord blood [6–9]. A study that examined 27 mother–newborn dyads found that pregnancy-related stress, assessed early in gestation, was associated with shorter telomere length in newborn cord blood [6]. In addition, two prior studies reported associations between higher maternal perceived stress and shorter newborn telomere length [8,9]. Lastly, a study of 24 mother-newborn dyads found that newborns whose mothers reported a higher level of psychosocial stress had significantly shorter telomere length compared to newborns of mothers with low stress [7]. All of the aforementioned studies were limited to one measure of maternal stress [6–9], and relied upon standard statistical approaches that make stringent modeling assumptions [6–8].

In this work, we evaluated the relationship between newborn telomere length and a comprehensive suite of chronic maternal stressors that included: financial strain, food insecurity, having a high-strain job, poor perceived neighborhood quality, low perceived social status in one’s community, experience of stressful or traumatic life events, caregiving for a dependent family member, global perception of current stress (perceived stress), and unplanned pregnancy. We used robust statistical methods that make use of modern machine learning algorithms to evaluate these relationships in the largest to-date cohort of economically and ethnically diverse pregnant women, taking into account a comprehensive set of potential confounders that includes race/ethnicity.

## Materials and methods

### Study population

Participants were part of the University of California, San Francisco (UCSF) Chemicals in Our Bodies (CIOB) study which recruited 510 pregnant women between 2014 and 2018 from two San Francisco hospitals where pregnant women seek prenatal care and delivery services: Zuckerberg San Francisco General Hospital (n = 189), which serves predominantly low-income women of color who do not have health insurance, and Moffitt-Long/Mission Bay Hospitals (n = 321), which serve an economically and ethnically diverse population of women who are primarily privately insured. English or Spanish-speaking mothers between 18 and 40 years of age with singleton pregnancies between 13 to 27 weeks of gestation (2^nd^ trimester) were eligible for enrollment. We were able to successfully collect umbilical cord blood at delivery from 355 participants. This subgroup constituted our study population. CIOB study protocols were approved by the Institutional Review Board of the University of California, San Francisco (Protocol # 13–12160). All study participants provided written, informed consent prior to participating in CIOB. Our study did not include minors.

### Measures of chronic stressors and psychosocial stress

Measures of maternal psychosocial stress were derived from answers to a stress assessment questionnaire that was administered by study personnel during a second trimester prenatal care visit. The stress assessment questionnaire was administered via interview in either English or Spanish, depending on participant preference. Questions were developed from the literature on factors related to psychosocial stress, work, social and physical environments and have been previously validated in other study populations and were piloted tested on a subset of women in CIOB. The interview questionnaire is provided as **Supporting Information**. We examined nine potential stressors, discussed in detail below. In addition, **S1 Table** lists the questions used to assess each stressor, their scoring and how scores were combined into the binary indicators of exposure to each stressor that were used in our analyses. The ascertainment timeframe for the individual stressors is provided within the questions in **S1 Table.**

#### Financial strain

Women were considered to be experiencing financial strain if their reported household income was below the poverty line for San Francisco County in 2017 [10], or if they answered “difficult/very difficult” to the question “How hard is it for you to pay for very basics like food, housing, medical care, and heating?”.

#### Food insecurity

We used the United States Department of Agriculture (USDA) measure of food insecurity [11–13]. Women were considered food-insecure if they answered “yes” to any of the following questions: “In the last 12 months, did you or other adults in your household ever cut the size of your meals or skip meals because there wasn't enough money for food?”, “In the last 12 months, did you ever eat less than you felt you should because there wasn't enough money to buy food?”, “In the last 12 months, were you ever hungry but didn't eat because you couldn't afford more food?” or if they answered “often/very often” to either of the questions: “The food that we bought just didn't last, and we didn't have money to get more”, “We couldn't afford to eat balanced and nutritious meals”.

#### High job strain

Participants were considered to experience high job strain if they reported working for pay and experiencing heavy demand and low control in their workplace [14]. Jobs were considered *high demand* if women strongly disagreed/disagreed with any of the statements “I am not asked to do an excessive amount of work”, “Considering my efforts and achievements, my pay is fair” or if they agreed/strongly agreed with the statement: “My job leaves me feeling too tired and stressed after work.” Jobs were considered *low control* if women strongly disagreed/disagreed with any of the statements: “My job allows me to make a lot of decisions on my own,” “I have an opportunity to develop my own special abilities”.

#### Poor neighborhood quality

Perceptions of participants’ neighborhood and social environments were assessed with a questionnaire adapted from a validated instrument [15–18] that combines measures of: (i) collective efficacy, (ii) neighborhood safety, (iii) neighborhood satisfaction, and (iv) physical order. Participants were exposed to poor neighborhood quality if they reported that their neighborhood lacked any of the four components.

Collective efficacy indicates cohesion among neighbors combined with their willingness to intervene on behalf of the common good [18]. Participants were asked how strongly they agreed (1 = strongly disagree, 2 = somewhat disagree, 3 = neither agree nor disagree, 4 = somewhat agree, 5 = strongly agree) that: (a) “people around here are willing to help their neighbors,” (b) “this is a close-knit neighborhood,” (c) “people in this neighborhood can be trusted,” (d) “people in this neighborhood generally don’t get along with each other,” (e) “people in this neighborhood do not share the same values”, (f) “children were skipping school and hanging out on a street corner”, (g) “children were spray-painting graffiti on a local building”, (h) “children were showing disrespect to an adult”, (i) “a fight broke out in front of their house”. Positively worded statements (a)-(c) were reverse scored (1 = strongly agree, …, 5 = strongly disagree). Women experienced low collective efficacy if their average score was ≥4.

Similarly, participants were considered to live in a disorderly neighborhood if their average score on the following three questions was ≥4: (a) “there is a lot of loud noise from cars, motorcycles, music, neighbors, or airplanes in my neighborhood”, (b) “my neighborhood has a lot of vacant lots or vacant houses”, (c) “there is heavy car or truck traffic in this neighborhood”. Women were considered to lack neighborhood safety if they strongly disagreed/disagreed that they felt safe in their neighborhood. Women were considered dissatisfied if they strongly disagreed/disagreed that their neighborhood is a good place to live, or agreed/strongly agreed that they would move out of their neighborhood if they could.

#### Low community standing

The subjective social status instrument was adapted from the MacArthur Scale of Social Status [19–21]. Participants were considered to have low perceived social standing in their community if they answered <5 when asked how would they would rank themselves on a scale from 1 to 10, with 1 representing people of lowest standing and 10 representing people of highest standing in their community.

#### Perceived stress

We used the 4-item Perceived Stress Scale (PSS-4) to assess the degree to which a participant perceived her life as uncontrollable, unpredictable, and overloading [22,23]. Women were asked how often (0 = never, 1 = rarely, 2 = sometimes, 3 = often, 4 = very often) they felt the following four symptoms: (1) unable to control important things in life, (2) confident in ability to handle personal problems, (3) things were going your way, (4) and difficulties were piling up so high that you could not overcome them. Answers to questions (2) and (3) were reverse scored and a total score of ≥9 denoted a high level of perceived stress [22–25].

#### Caregiving for a dependent family member

Participants were considered to have experienced stress due to caregiving for a dependent if they answered often/very often to any of the questions [26,27]: “How often in the past 5 years were you responsible for the care and well-being of a parent or any older relative?”, “How often in the past 5 years were you responsible for the care and well-being of a child who needs or uses more medical care, mental health, or educational services than is usual for most children of the same age?”.

#### Stressful/Traumatic life events

Participants were asked whether they had experienced any of the following in the 12 months prior to the interview: (1) a close family member was very sick and had to go to the hospital, (2) she was separated or divorced from her husband or partner, (3) she moved to a new address, (4) her spouse lost his or her job, (5) she lost her job even though she wanted to go on working, (6) she argued with her spouse or partner more than usual, (7) the spouse or partner didn’t want her to be pregnant, (8) she had a lot of bills she couldn’t pay, (9) she was in a physical fight, (10) her spouse or partner had serious legal problems, (11) someone close to her had a problem with drinking or drugs, (12) someone close to her died, (13) she or a close family member had experienced immigration problems. Experience of two or more events was considered a high level of stress [28,29].

#### Unplanned pregnancy

Participants were considered to have an unplanned pregnancy if they answered “Didn’t want to be pregnant then” or “I wanted to be pregnant later” to the question: “Thinking back to just before you got pregnant, how did you feel about becoming pregnant?” Possible answers were: “Didn’t want to be pregnant then”, “I wanted to be pregnant then”, “I wanted to be pregnant later”, “I wanted to be pregnant sooner” [30].

### Outcome

Telomere length was measured in the newborn’s umbilical cord leukocytes collected at delivery by labor and delivery clinicians. Methods for measuring newborn telomere length applied a well-established assay [31] described in detail elsewhere [27,32]. Briefly, total genomic DNA was isolated from whole blood samples. Average telomere length values were measured by a quantitative polymerase chain reaction assay that determines the relative ratio of telomere repeat abundance to single-copy gene abundance (T/S ratio) in experimental samples as compared with a reference DNA sample. Samples were measured twice, each with triplicate wells. The average inter-assay coefficient of variation (CV) was 1.9% for this study and the intra-class correlation coefficient (ICC) was 98%. The variance within and among plates was 0.00 and 0.06, respectively. The T/S ratio was approximately normally distributed.

### Statistical analysis

Differences between the distribution of variables across race/ethnicity categories were assessed using one-way Analysis of Variance (ANOVA) for continuous variables and Fisher's exact test for categorical ones. A correlation matrix of the stressors and covariates is visualized in **S1 Fig**.

We used Targeted Minimum Loss-Based Estimation (TMLE) to assess the independent association of each stressor with newborn telomere length accounting for all other stressors and covariates (*joint effects*). As a sensitivity analysis, we evaluated the effect of each stressor controlling for covariates but not the remaining stressors (*individual effects*, **S2 Table**). We also examined the associations between maternal stressors and newborn telomere length stratified by infant sex and race/ethnicity (White/Women of Color). Hispanic/Latina, Black, Asian, and Other were combined into one “Women of Color” category due to small sample sizes of each of these demographic groups. We additionally estimated joint and individual effects using linear regression (reporting β coefficients) so that our findings could be compared with prior studies, all of which used standard regression. While traditional regression requires that a correctly specified model be chosen *a priori*, the exposure and outcome models for TMLE can be modelled non-parametrically using SuperLearner. SuperLearner is a machine-learning approach that uses cross-validation, which protects against overfitting [33], to create the best combination of algorithm-specific estimates from a library of algorithms, thereby avoiding the potential bias caused by assuming a fixed parametric relationship between covariates [34,35]. Our library included Random Forest, Generalized linear models (GLMs) main terms only and with interactions, stepwise GLMs, GLMs with Elastic Net Regularization, Generalized Additive Models, and Gradient Boosting Machines with regression and classification trees. In addition to the aforementioned advantage, TMLE is consistent under partial model misspecification (double robustness property), and is efficient when models for the exposure and outcome are correctly specified (local efficiency) [36–40]. Additional details about the use and application of TMLE in observational health studies are available elsewhere [41].

Linear regression estimates a beta coefficient for the average treatment effect. TMLE also estimates the average treatment effect, but applies machine learning to more flexibly model covariates. Both model estimates are interpreted as the average change in t/s ratio associated with high stressor exposures. All analyses were adjusted for maternal age, education, parity, race/ethnicity, and hospital of delivery. Mother’s race/ethnicity (categorized as Non-Hispanic White, Hispanic/Latina, African-American, Asian/Pacific-Islander, or other/mixed/unknown) and education were self-reported at enrollment. Maternal age and parity were obtained from the medical record. We applied the Benjamini-Hochberg (BH) correction to control the False Discovery Rate [42]. The analysis was performed using the *tmle* package [43] in R [44].

## Results

The mean age at enrollment for women in our study population was 33 years; 44% of mothers were White, 28% were Latina, 20% were Asian, and 5% were Black; 72% were college graduates or held graduate degrees (**Table 1**). A greater proportion of Latina mothers reported financial strain, food insecurity, or high job strain than other racial/ethnic groups (**Table 1**). A greater proportion of Black mothers reported poor neighborhood quality, experiencing stressful/traumatic life events, or having an unplanned pregnancy than other racial/ethnic groups (**Table 1**). There were no differences in newborn telomere length by race/ethnicity (**Table 1**).

**Table 1:** Characteristics of the UCSF Chemicals in Our Bodies (CIOB) study population.

		Race/Ethnicity				
	Overall (N = 355)	White (N = 156)	Hispanic/Latina (N = 100)	Asian (N = 72)	Black (N = 17)	P *
Maternal stressor, *n (%)*						
Financial strain	104 (29.3)	14 (9)	61 (61)	19 (26.4)	8 (47.1)	<0.001
Food insecurity	33 (9.3)	3 (1.9)	20 (20)	5 (6.9)	3 (17.6)	<0.001
High job strain	31 (8.7)	3 (1.9)	22 (22)	5 (6.9)	1 (5.9)	<0.001
Poor neighborhood quality	66 (18.6)	20 (12.8)	31 (31)	9 (12.5)	5 (29.4)	<0.001
Low community standing	34 (9.6)	9 (5.8)	17 (17)	5 (6.9)	2 (11.8)	0.04
High level of perceived stress	36 (10.1)	14 (9)	13 (13)	5 (6.9)	3 (17.6)	0.46
Caregiving for a dependent	48 (13.5)	8 (5.1)	23 (23)	13 (18.1)	4 (23.5)	<0.001
Stressful/traumatic events	195 (54.9)	71 (45.5)	69 (69)	39 (54.2)	13 (76.5)	<0.001
Unplanned pregnancy	78 (22)	23 (14.7)	31 (31)	16 (22.2)	7 (41.2)	<0.001
Educational attainment, *n (%)*					
Less than high school	27 (7.6)	0 (0)	24 (24)	1 (1.4)	2 (11.8)	0.04
High school/some college	74 (20.8)	13 (8.3)	41 (41)	10 (13.9)	6 (35.3)	
Bachelor’s degree	100 (28.2)	53 (34)	16 (16)	27 (37.5)	4 (23.5)	
Graduate degree	154 (43.4)	90 (57.7)	19 (19)	34 (47.2)	5 (29.4)	
Maternal age, *Mean ±SD*	33.4 ± 4.8	34.5 4.3	31.25 ± 5.7	34.35 ± 3.6	31.94 ± 5.2	<0.001
Gestational age, *Mean ± SD*	39.0 ± 1.6	39.19 ± 1.5	38.74 ± 1.5	38.79 ± 1.8	38.76 ± 2	0.17
T/S, *Mean ± SD*	1.5 ± 0.2	1.5 ± 0.3	1.5 ± 0.2	1.5 ± 0.2	1.4 ± 0.2	0.49

*Racial/Ethnic differences in the distribution of continuous variables were assessed using one-way Analysis of Variance (ANOVA); differences in the distribution of categorical variables were assessed using the Fisher's exact test.

Note: 10 participants were classified as “other/multi-racial” race/ethnicity and the distribution of stress and demographics were not provided for these women due to small N.

Abbreviations: SD, standard deviation.

TMLE and linear regression estimates denote the change in T/S ratio associated with exposure to each stressor. When joint effects were examined, none of the maternal stressors were associated with newborn telomere length in linear regression analyses (**Table 2**). Low community standing (-0.09; 95% confidence interval [CI] -0.19 to 0.00) and perceived stress (-0.07; 95% CI -0.15 to 0.01) had the strongest negative associations with newborn telomere length in TMLE analyses, with multiple comparison-adjusted P values above the significance threshold (0.29 and 0.29 respectively, **Table 2**). Poor neighborhood quality was modestly associated with shorter newborn telomere length (-0.07; 95% CI -0.17 to 0.002), although findings were attenuated after multiple comparison adjustment (**Table 2**). None of the maternal stressors were associated with shorter newborn telomere length in either linear regression or TMLE analyses when stressors were examined individually (**S2 Table**).

**Table 2:** Joint measures of associations between maternal stressors during pregnancy and newborn telomere length: Linear regression and Targeted Minimum Loss-based Estimation (TMLE) results.

	Linear regression			TMLE		
	Estimate* (95% CI)	Uncorrected P value	P value**	Estimate* (95% CI)	Uncorrected P value	P value**
Financial strain	0.02 (-0.06, 0.09)	0.69	0.82	0.03 (-0.08, 0.13)	0.63	0.71
Food insecurity	0.11 (0.00, 0.21)	0.04	0.39	0.05 (-0.02, 0.11)	0.20	0.36
High job strain	0.04 (-0.06, 0.13)	0.47	0.82	0.04 (-0.01, 0.10)	0.10	0.29
Poor neighborhood quality	-0.03 (-0.10, 0.05)	0.49	0.82	-0.07 (-0.17, 0.02)	0.14	0.31
Low community standing	-0.08 (-0.17, 0.01)	0.09	0.53	-0.09 (-0.19, 0.00)	0.06	0.29
High level of perceived stress	-0.03 (-0.11, 0.06)	0.52	0.82	-0.07 (-0.15, 0.01)	0.08	0.29
Caregiving for a dependent	0.04 (-0.04, 0.12)	0.30	0.82	0.03 (-0.05, 0.11)	0.48	0.71
Stressful/traumatic events	0.01 (-0.05, 0.06)	0.79	0.82	0.01 (-0.05, 0.07)	0.75	0.75
Unplanned pregnancy	-0.01 (-0.07, 0.06)	0.82	0.82	-0.02 (-0.09, 0.05)	0.55	0.71

*Linear regression estimates a beta coefficient for the average treatment effect. TMLE also estimates the average treatment effect, but applies machine learning to more flexibly model covariates. Both estimates are interpreted as the average change in t/s ratio associated with high stressor exposures.

**Benjamini-Hochberg p-value.

Models adjusted for maternal age, education, parity, race/ethnicity, and delivery hospital.

To assess potential different effects by maternal race/ethnicity, we used TMLE to estimate joint stressor effects separately for White women (n = 156) and Women of Color (n = 189). High job strain was associated with longer newborn telomere length among White women (0.12; 95% CI 0.07 to 0.16) and shorter length among Women of Color (0.01; 95% CI -0.08 to 0. 11; **S3 Table**). Perceived stress was associated with shorter newborn telomere length among White women (-0.17; 95% CI -0.37 to 0.01) but not among Women of Color (0.03; 95% CI -0.06 to 0.12; **S3 Table**).

To assess potential different effects by newborn sex, we estimated joint stressor effects using TMLE for male (N = 173) and female (N = 194) newborns separately (**S4 Table**). High level of perceived stress and unplanned pregnancy were associated with shorter telomere length among females (perceived stress: -0.12; 95% CI –0.22 to -0.02 and unplanned pregnancy: -0.08; 95% CI –0.20 to 0.04) but longer among males (perceived stress: 0.03; 95% CI –0.07 to 0.13 and unplanned pregnancy: 0.02; 95% CI –0.06 to 0.10). We observed stronger associations with food insecurity for male newborns (0.07; 95% CI 0.03 to 0.11) compared to female newborns (-0.05; 95% CI –0.15 to 0.06). In contrast, low community standing had stronger associations with shorter newborn telomeres among males (-0.14; 95% CI –0.24 to –0.03) compared with females (-0.05; 95% CI –0.16 to 0.06). Upon adjustment for multiple comparisons, all associations were attenuated with the exception of the food insecurity association among males.

## Discussion

In a diverse population of pregnant women receiving prenatal and delivery services at two San Francisco hospitals, we found that a greater proportion of Latina mothers reported financial strain, food insecurity, and high job strain, while a greater proportion of Black mothers reported poor neighborhood quality, experiencing stressful/traumatic life events, or having an unplanned pregnancy than other racial/ethnic groups. We also found that high levels of perceived stress and low perceived community standing were associated with shorter newborn telomere length, but the associations were not statistically significant after correcting for multiple comparisons.

Our findings add to a growing body of evidence that self-reported prenatal psychosocial stress is associated with shorter newborn telomere length [6–9]. Our study, which is larger and includes a more diverse population than prior studies [6–9], confirms previous findings of an association between shorter newborn telomere length and maternal perceived stress [8]. To our knowledge, no other studies have examined newborn telomere length with the multiple stressors that we considered here. Our flexible modeling approach, correction for multiple testing, and consideration of joint effects differ from previous approaches, and may account for some differences in findings. Suspecting potentially different effects by maternal race/ethnicity and infant sex, we conducted analyses separately for White women and Women of Color, and male and female newborns. Our results are suggestive of potentially differential effects of maternal stressors by race/ethnicity, particularly with respect to job strain and perceived stress. Stratification by newborn sex suggests differential effects for financial strain, perceived stress, and unplanned pregnancy. However, these findings need to be examined further in larger, better powered studies.

Potential limitations of our study should be considered in the interpretation of our findings. We did not have information on paternal age, which has been associated with telomere length in children in several studies [3]; however, we did control for maternal age, which is typically highly correlated with paternal age. In addition, information on maternal and paternal telomere length was not available for our study population. While the ascertainment window for all stressors spanned the first trimester, maternal stress during the third trimester was not reported and we were unable to assess whether self-reported stressors were consistent throughout the index pregnancy. Only a small number of Black women (n = 17) were included in our sample, which limited our ability to examine associations between maternal stressors and telomere length across specific racial and ethnic groups. Lastly, newborn telomere length was measured in cord blood, which may be subject to contamination from maternal blood as this has been shown in studies of DNA methylation done in cord blood [45].

In addition to the aforementioned limitations, this work has several strengths. To our knowledge, this study is the largest to date to evaluate the relationship between multiple maternal stressors and newborn telomere length. Our heterogeneous population included an ethnically and economically diverse group of women, increasing the generalizability of our results. Furthermore, our approach measured maternal psychosocial stress in multiple domains to better understand what sources of stress are most influential. Both individual and joint effects were evaluated. The use of cross-validated ensemble learners improved model fit and reduced bias due to model misspecification, allowing us to establish associations not observed with traditional regression approaches.

Given the higher burden of psychosocial stressors among Women of Color, our results could inform intervention strategies that seek to address underlying causes and coping strategies related to maternal social stressors during pregnancy, particularly among marginalized population groups.

## Supporting information

S1 Fig Correlation matrix of all stressors and covariates. (DOCX)

S1 Table Summary of questions included in the assessment of each stressor and their scoring. (DOCX)

S2 Table Linear regression and Targeted Minimum Loss-based Estimation (TMLE) measures of individual associations between each maternal stressor during pregnancy and newborn telomere length. (DOCX)

S3 Table Targeted Minimum Loss-based Estimation (TMLE) joint measures of associations between maternal stressors during pregnancy and newborn telomere length, by race/ethnicity. (DOCX)

S4 Table Targeted Minimum Loss-based Estimation (TMLE) measures of joint associations between maternal stressors during pregnancy and newborn telomere length, by infant sex. (DOCX)

S1 File (PDF)
