# Supplementary material for: The association of maternal psychosocial stress with newborn telomere length
Source: PLoS One. 2020 Dec 10;15(12):e0242064. doi: 10.1371/journal.pone.0242064 (PMC7728273; doi:10.1371/journal.pone.0242064)
Supplement: S1 Fig — (DOCX) [file pone.0242064.s001.docx]

###

**Figure S1.** Correlation matrix of all stressors and covariates.
