# Supplementary material for: The association of maternal psychosocial stress with newborn telomere length
Source: PLoS One. 2020 Dec 10;15(12):e0242064. doi: 10.1371/journal.pone.0242064 (PMC7728273; doi:10.1371/journal.pone.0242064)
Supplement: S1 Table — (DOCX) [file pone.0242064.s002.docx]

| **Table S1**. Summary of questions included in the assessment of each stressor and their scoring. | |
| --- | --- |
| **Stress Category/***Potential Answers/*Questions | Scoring |
| **Financial strain** |  |
| A.16. What is the combined family income, before taxes? |  |
| A.23. How hard is it for you to pay for very basics like food, housing, medical care, and heating?  *(1: Not difficult at all; 2: Not very difficult; 3: Somewhat difficult; 4: Very difficult.)* | 1-4 |
| **Household income (A.16) below 2017 San Francisco county poverty line OR A.23 >= 3.** |  |
| **Food insecurity** |  |
| *How often did this happen in your household in the last 12 months (1: Never True; 2: Sometimes True; 3: Often True).* |  |
| C.82. "The food that we bought just didn't last, and we didn't have money to get more." | 1-3 |
| C.83. "We couldn't afford to eat balanced and nutritious meals." | 1-3 |
| *Did any of the following happen in your household in the last 12 months (0: No; 1: Yes).* |  |
| C.84. Adults ever cut or skip meals because there wasn't enough money for food? | 0-1 |
| C.86. Did you ever eat less than you felt you should because there wasn't enough money to buy food? | 0-1 |
| C.87. Were you ever hungry but didn't eat because you couldn't afford more food? | 0-1 |
| **C.82 >= 2 OR C.83 >= 2 OR C.84 + C.86 + C.87 > 0.** |  |
| **Having a high strain job** |  |
| C.1. Are you currently working for pay, looking for work, retired, a homemaker or raising children full time, a student, or something else? |  |
| *Thinking about your current job, how likely is each of the statements (1: Very unlikely; 2: Unlikely; 3: Neither likely nor unlikely; 4: Likely; 5: Very likely).* |  |
| C.6. My job allows me to make a lot of decisions on my own. | 1-5 |
| C.7. I have an opportunity to develop my own special abilities. | 1-5 |
| C.8. I am not asked to do an excessive amount of work. | 1-5 |
| C.9. My job leaves me feeling too tired and stressed after work. | 1-5 |
| C.10. Considering my efforts and achievements, my salary (pay) is fair. | 1-5 |
| **High Demand: C.8 in [1,2] OR C.9 in [4,5] OR C.10 in [1,2]** |  |
| **Low Control: C.6 in [1,2] OR C.7 in [1,2]** |  |
| **"Currently working for pay" AND (high demand AND low control).** |  |

| **Perceived poor neighborhood quality** |  |
| --- | --- |
| *Social Cohesion  (1. Strongly disagree; 2: Somewhat disagree; 3: Neither agree nor disagree; 4: Somewhat agree; 5: Strongly agree)* |  |
| C.47. People around here are willing to help their neighbors. | 5-1 |
| C.50. This is a close-knit neighborhood. | 5-1 |
| C.49. People in this neighborhood can be trusted. | 5-1 |
| C.53. People in this neighborhood generally don't get along with each other. | 1-5 |
| C.54. People in this neighborhood don't share the same values. | 1-5 |
| *Informal Social Control* |  |
| C.57. Children were skipping school and hanging out on a street corner | 1-5 |
| C.58. Children were spray-painting graffiti on a local building | 1-5 |
| C.59. Children were showing disrespect to an adult | 1-5 |
| C.60. A fight broke out in front of their house | 1-5 |
| *Neighborhood Safety* |  |
| C.48. I feel safe in this neighborhood. | 5-1 |
| *Neighborhood Satisfaction* |  |
| C.46. I think this neighborhood is a good place for me to live. | 5-1 |
| C.52 I would move out of this neighborhood if I could. | 1-5 |
| *Physical Disorder* |  |
| C.55. There is a lot of loud noise from cars, motorcycles, music, neighbors, or airplanes in my neighborhood. | 1-5 |
| C.56. My neighborhood has a lot of vacant lots or vacant houses. | 1-5 |
| C.51. There is heavy car or truck traffic in this neighborhood. | 1-5 |
| **Low collective efficacy: Average of C.47 to C.60 >= 4.** |  |
| **Find neighborhood unsafe: C.48 >= 4.** |  |
| **Dissatisfied with neighborhood: Average of C.46 and C.52 >= 4.** |  |
| **Disorderly neighborhood: Average of C.51, C.55, C.56 >= 4.** |  |
| **Low collective efficacy OR unsafe OR dissatisfied OR disorderly.** |  |
| **Perceived low standing in one's community** |  |
| C.80. Where would you place yourself today, on a scale of 1 (lowest) - 10 (highest) standing in your community. |  |
| **C.80 <= 4.** |  |

| **Global perception of current stress (Perceived stress)** |  |
| --- | --- |
| *How often (0: Never; 1: Rarely; 2: Sometimes; 3: Often; 4: Very often) in the past 5 years have you felt:* |  |
| C.16. That you were unable to control the important things in your life? | 0-4 |
| C.17. Confident about your ability to handle your personal problems? | 4-0 |
| C.18. That things were going your way? | 4-0 |
| C.19. That difficulties were piling up so high that you could not overcome them? | 0-4 |
| **C.16 + C.17 + C.18 + C.19 >= 9.** |  |
| **Caring for a dependent family member** |  |
| *How often in the past 5 years  1: Never - 5: Very Frequently - 1: Never; 2: Rarely; 3: Sometimes; 4: Often; 5: Very often.* |  |
| C.21. Were you responsible for the care and well-being of a parent or any older relative? | 1-5 |
| C.22. Were you responsible for a child who needs more medical/health/educational services? | 1-5 |
| **C.21 in [4, 5] OR C.22 in [4, 5].** |  |
| **Experiencing stressful or traumatic life events** |  |
| *Has any of the following happened during the last 12 months (1: Yes; 0: No).* |  |
| C.29. A close family member was very sick and had to go into the hospital | 0-1 |
| C.30. I got separated or divorced from my husband or partner | 0-1 |
| C.31. I moved to a new address | 0-1 |
| C.32. My spouse or partner lost his/her job | 0-1 |
| C.33. I lost my job even though I wanted to go on working | 0-1 |
| C.34. I argued with my spouse or partner more than usual | 0-1 |
| C.35. My spouse or partner said s/he didn't want me to be pregnant | 0-1 |
| C.36. I had a lot of bills I couldn't pay | 0-1 |
| C.37. I was in a physical fight | 0-1 |
| C.38. My spouse or partner had serious legal problems | 0-1 |
| C.39. Someone very close to me had a problem with drinking or drugs | 0-1 |
| C.40. Someone very close to me died | 0-1 |
| C.40.a I or a close family member has experienced immigration problems | 0-1 |
| **Sum (C.29 to C.40a) >= 2.** |  |
| **Unplanned pregnancy** |  |
| *1: I didn't want to be pregnant then; 2: I wanted to be pregnant then; 3: I wanted to be pregnant later; 4: I wanted to be pregnant sooner.* |  |
| B.2. Thinking back to just before you got pregnant, how did you feel about becoming pregnant? | 1-4 |
| **B.2 in [1, 3].** |  |
