# Supplementary material for: The association of maternal psychosocial stress with newborn telomere length
Source: PLoS One. 2020 Dec 10;15(12):e0242064. doi: 10.1371/journal.pone.0242064 (PMC7728273; doi:10.1371/journal.pone.0242064)
Supplement: S2 Table — (DOCX) [file pone.0242064.s003.docx]

### **Table S2.** Linear Regression and Targeted Minimum Loss-based Estimation (TMLE) measures of individual associations between each maternal stressor during pregnancy and newborn telomere length.

|  | Linear Regression | | |  | TMLE | | | |  |
| --- | --- | --- | --- | --- | --- | --- | --- | --- | --- |
|  | Estimate (95% CI) | | P value* |  | Estimate (95% CI) | | P value* | |  |
| Financial strain | 0.04 (-0.04, 0.11) | | 0.34 |  | 0.03 (-0.03, 0.08) | | | 0.37 |  |
| Food insecurity | 0.08 (-0.01, 0.17) | | 0.08 |  | 0.06 (0.01, 0.11) | | | 0.01 |  |
| High job strain | 0.03 (-0.06, 0.12) | | 0.53 |  | 0.03 (-0.03, 0.08) | | | 0.36 |  |
| Poor neighborhood quality | -0.01 (-0.08, 0.06) | | 0.81 |  | -0.04 (-0.12, 0.05) | | | 0.38 |  |
| Low community standing | -0.06 (-0.14, 0.03) | | 0.21 |  | -0.09 (-0.17, -0.01) | | | 0.03 |  |
| High level of perceived stress | 0.00 (-0.09, 0.08) | | 0.93 |  | -0.03 (-0.12, 0.06) | | | 0.55 |  |
| Caregiving for a dependent | 0.04 (-0.03, 0.12) | | 0.28 |  | 0.06 (-0.02, 0.13) | | | 0.15 |  |
| Stressful/traumatic events | 0.01 (-0.04, 0.07) | | 0.60 |  | 0.01 (-0.04, 0.06) | | | 0.72 |  |
| Unplanned pregnancy | 0.00 (-0.07, 0.06) | | 0.90 |  | -0.01 (-0.08, 0.06) | | | 0.82 |  |
|  |  |  |  |  |  |  |  | |  |

*Benjamini-Hochberg p-value.

Adjusted for maternal age, education, parity, race/ethnicity, and delivery hospital.
