# Supplementary material for: The association of maternal psychosocial stress with newborn telomere length
Source: PLoS One. 2020 Dec 10;15(12):e0242064. doi: 10.1371/journal.pone.0242064 (PMC7728273; doi:10.1371/journal.pone.0242064)
Supplement: S3 Table — (DOCX) [file pone.0242064.s004.docx]

### **Table S3**. Targeted Minimum Loss-based Estimation (TMLE) joint measures of associations between maternal stressors during pregnancy and newborn telomere length, by race/ethnicity.

|  | White Women | | |  | Women of Color | | |  |
| --- | --- | --- | --- | --- | --- | --- | --- | --- |
|  | Estimate (95% CI) | | P value* |  | Estimate (95% CI) | | P value* |  |
| Financial strain | -0.03 (-0.14, 0.07) | | 0.74 |  | -0.01 (-0.08, 0.06) | | 0.80 |  |
| Food insecurity | -0.01 (-0.06, 0.03) | | 0.74 |  | 0.02 (-0.07, 0.10) | | 0.80 |  |
| High job strain | 0.12 (0.07, 0.16) | | 0.00 |  | 0.01 (-0.08, 0.11) | | 0.80 |  |
| Poor neighborhood quality | -0.12 (-0.38, 0.14) | | 0.66 |  | -0.09 (-0.17, -0.01) | | 0.23 |  |
| Low community standing | -0.07 (-0.20, 0.05) | | 0.56 |  | -0.10 (-0.20, 0.01) | | 0.30 |  |
| High level of perceived stress | -0.18 (-0.37, 0.01) | | 0.17 |  | 0.03 (-0.06, 0.12) | | 0.77 |  |
| Caregiving for a dependent | 0.15 (0.00, 0.30) | | 0.17 |  | -0.04 (-0.14, 0.06) | | 0.77 |  |
| Stressful/traumatic events | 0.01 (-0.07, 0.10) | | 0.80 |  | 0.03 (-0.05, 0.10) | | 0.77 |  |
| Unplanned pregnancy | -0.02 (-0.15, 0.11) | | 0.80 |  | -0.03 (-0.10, 0.05) | | 0.77 |  |
|  |  |  |  |  |  |  |  |  |

*Benjamini-Hochberg p-value.

Adjusted for maternal age, education, parity, delivery hospital, and race/ethnicity (for women of color).
