# Supplementary material for: The association of maternal psychosocial stress with newborn telomere length
Source: PLoS One. 2020 Dec 10;15(12):e0242064. doi: 10.1371/journal.pone.0242064 (PMC7728273; doi:10.1371/journal.pone.0242064)
Supplement: S4 Table — (DOCX) [file pone.0242064.s005.docx]

### **Table S4**. Targeted Minimum Loss-based Estimation (TMLE) measures of joint associations between maternal stressors during pregnancy and newborn telomere length, by infant sex.

|  | Male | | |  | Female | | |  |
| --- | --- | --- | --- | --- | --- | --- | --- | --- |
|  | Estimate (95% CI) | | P value* |  | Estimate (95% CI) | | P value* |  |
| Financial strain | -0.01 (-0.09, 0.07) | | 0.94 |  | 0.12 (0.00, 0.24) | | 0.14 |  |
| Food insecurity | 0.07 (0.03, 0.11) | | 0.01 |  | -0.05 (-0.15, 0.06) | | 0.51 |  |
| High job strain | -0.02 (-0.12, 0.08) | | 0.94 |  | 0.07 (0.01, 0.13) | | 0.09 |  |
| Poor neighborhood quality | -0.05 (-0.21, 0.12) | | 0.94 |  | -0.07 (-0.18, 0.04) | | 0.42 |  |
| Low community standing | -0.14 (-0.24, -0.03) | | 0.06 |  | -0.05 (-0.16, 0.06) | | 0.51 |  |
| High level of perceived stress | 0.03 (-0.07, 0.13) | | 0.94 |  | -0.12 (-0.22, -0.02) | | 0.09 |  |
| Caregiving for a dependent | 0.01 (-0.09, 0.12) | | 0.94 |  | 0.03 (-0.09, 0.14) | | 0.62 |  |
| Stressful/traumatic events | 0.00 (-0.08, 0.07) | | 0.94 |  | 0.03 (-0.06, 0.12) | | 0.62 |  |
| Unplanned pregnancy | 0.02 (-0.06, 0.10) | | 0.94 |  | -0.08 (-0.20, 0.04) | | 0.42 |  |
|  |  |  |  |  |  |  |  |  |

*Benjamini-Hochberg p-value.

Adjusted for maternal age, education, parity, race/ethnicity, and delivery hospital.
